# Management of Mycosis Fungoides With Chlormethine Hydrochloride Gel in Combination With Systemic Therapies: A Case Series

**DOI:** 10.1155/crdm/8813008

**Published:** 2026-02-09

**Authors:** Gabor Dobos, Constanze Jonak, Kai-Christian Klespe, Marion Wobser, Adèle de Masson, Johanna Hoffmann, Christina Mitteldorf

**Affiliations:** ^1^ Freie Universität Berlin and Humboldt-Universität zu Berlin, Skin Cancer Center HTCC, Charité-Universitätsmedizin Berlin, Berlin, Germany, charite.de; ^2^ Department of Dermatology, Medical University of Vienna, Vienna, Austria, meduniwien.ac.at; ^3^ Department of Dermatology, Allergology and Venereology, Hannover Medical School, Hannover, Germany, mh-hannover.de; ^4^ Department of Dermatology and Allergology, University Hospital RWTH Aachen, Aachen, Germany, ukaachen.de; ^5^ Department of Dermatology, Venereology and Allergology, Würzburg University Hospital, Würzburg, Germany, ukw.de; ^6^ Department of Dermatology, Saint-Louis University Hospital, AP-HP, Université Paris Cité, Paris, France; ^7^ Department of Dermatology, Venereology and Allergology, University Medical Center Göttingen, Göttingen, Germany, umg.eu

**Keywords:** case report, chlormethine hydrochloride, combination therapy, mechlorethamine, mycosis fungoides, T-cell lymphoma

## Abstract

**Background:**

Mycosis fungoides (MF) is a subtype of T‐cell lymphoma that is characterised by the infiltration of malignant T cells into the skin. Treatment approaches usually include skin‐directed therapies for early‐stage disease and, additionally, systemic therapies for advanced stages. Chlormethine hydrochloride gel is recommended as a first‐line treatment option for adult patients with MF, with previous studies demonstrating its efficacy. Due to the rarity of this disease, available literature on the optimal treatment of MF with chlormethine gel is limited, creating challenges for making informed clinical decisions. Thus, we share our individual clinical experiences of selected patients on chlormethine combination treatments to increase the real‐world evidence for chlormethine gel in patients with MF.

**Case Presentation:**

We present the cases of five male and two female Caucasian patients above the age of 48 with Stage I–IV MF, presenting with symptoms of skin plaques and lesions. All patients were treated with chlormethine hydrochloride gel in combination with other skin‐directed and systemic therapies, including bexarotene, methotrexate, topical steroids, extracorporeal photopheresis, donor lymphocyte infusion and interferon‐α (IFN‐α) 2a. In most cases, chlormethine combination treatment resulted in disease control, e.g., plaque reduction, stable disease, and partial or complete response. The combination regimens were generally well tolerated, with associated adverse events being inflammation, pruritus and erythema.

**Conclusions:**

This case series reports on the efficacy and safety of chlormethine hydrochloride gel in combination with other topical and systemic therapies in reducing the skin lesion severity in patients with Stage I–IV MF in different real‐world settings.

## 1. Introduction

Mycosis fungoides (MF) is a subtype of cutaneous T‐cell lymphoma that represents approximately 50% of all primary cutaneous lymphomas [1, 2]. It is largely a disease of the adult population, with diagnosis generally at the age of 50 years or older, and typically follows a protracted and indolent disease course, particularly in early‐stage disease [2–4]. MF is characterised by the infiltration of malignant T cells into the skin [3]. Four clinical stages have been defined, with progression from localised macules (patches) and infiltrated plaques to tumours that may ulcerate, and eventually erythroderma. Consequently, infections and/or systemic spread may result in death [2, 5, 6].

Management approaches for MF are usually stage‐oriented and focussed on skin‐directed treatments with topical steroids, ultraviolet (UV) phototherapy, radiation therapy and the topical cytostatic agent chlormethine for early‐stage disease, with additional systemic therapies for advanced‐stage disease [5]. Therapeutic strategies aim to treat local lesions, prevent disease progression and maintain quality of life (QoL). However, despite treatment, lesions may be highly symptomatic, causing pain and itching for years to decades, which may severely affect patients’ daily functioning, QoL and ability to work [7–9].

Chlormethine, also known as mechlorethamine, is a bifunctional alkylating agent that inhibits rapidly proliferating cells and may interact with immune effectors [10, 11]. Chlormethine hydrochloride is recommended as a first‐line treatment option for adult patients with MF [6, 7]. This agent was initially developed in aqueous and ointment formulations, which presented challenges with preparation and application [7]. More recently, a topical 0.02% gel formulation was developed specifically for the treatment of MF that is stable and quick drying and can be administered at home by the patients or caregivers [7, 12]. According to the Summary of Product Characteristics (SmPC), a thin film of chlormethine 160 μg/g gel should be applied once daily to affected areas of the skin [12].

Chlormethine hydrochloride gel was approved in the USA in 2013 for the topical treatment of Stage IA and IB MF in patients who have received prior skin‐directed therapy [13], and in the EU in 2017 for the treatment of adult patients with MF of any stage [12], based on data from the randomised, multicentre study 201 [12, 14]. Study 201 (*N* = 260) demonstrated that chlormethine hydrochloride gel was noninferior to equal‐strength chlormethine ointment in patients with Stages IA, IB and IIA MF who had received at least one prior skin‐directed therapy. In addition, the study reported higher response rates for chlormethine hydrochloride gel versus ointment for the Composite Assessment of Index Lesion Severity (CAILS) scale (77% vs. 59%, respectively) and the Modified Severity‐Weighted Assessment Tool (mSWAT; 63% vs. 56%, respectively) in the efficacy evaluable population [12, 14].

Skin‐related adverse events (AEs) were documented in 62% of patients receiving chlormethine hydrochloride gel in the study, including skin irritation, pruritus, erythema, contact dermatitis, hyperpigmentation and folliculitis [14]. Treatment‐limiting skin reactions were reported in 20.3% of patients, and the large majority occurred within the first few months of treatment; no serious treatment‐related AEs were reported [14].

Insights into clinical decision‐making of healthcare professionals managing patients with MF who receive chlormethine hydrochloride gel therapy are limited in the literature due to the rarity of this disease, which presents a challenge for clinicians who are unfamiliar with this treatment option [10].

Here, we report a case series of seven patients with Stage I–IV MF who received chlormethine hydrochloride gel for the management of their disease in combination with other skin‐directed and systemic therapies. Our aim is to present our clinical experience to provide real‐world guidance on the management of MF and to illustrate the efficacy of chlormethine hydrochloride gel in treating MF lesions.

## 2. Case Presentation

### 2.1. Case 1

Case 1 documents a 68‐year‐old Caucasian male with skin plaques and a history of hypertension and dyslipidaemia, who received a diagnosis of MF (T2bN0M0B0) Stage IB in 2014, according to the International Society for Cutaneous Lymphomas (ISCL) and the European Organisation of Research and Treatment of Cancer (EORTC) staging system [15].

In June 2019, following 30 sessions of phototherapy, he started treatment with chlormethine hydrochloride 160 μg/g gel 5 days/week based on the decision of his treating doctor, which was increased to 7 days/week after 4 months. He achieved stable disease in November 2019 and commenced subcutaneous methotrexate 15 mg/week in addition to chlormethine hydrochloride. The methotrexate dose was increased to 20 mg/week in March 2021 and to 25 mg/week in September 2021, in combination with chlormethine hydrochloride gel 7 days/week.

**FIGURE 1:**
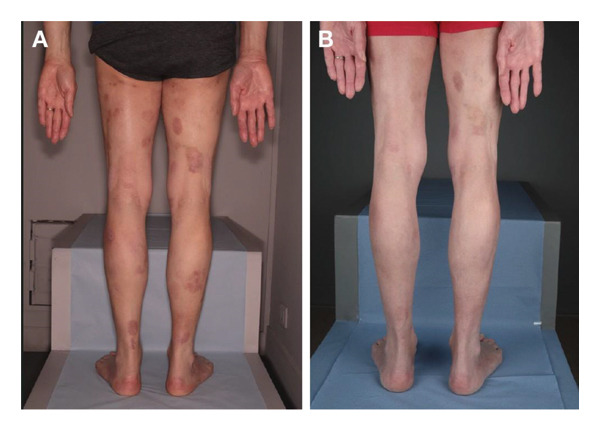
Case 1 clinical images. (A) Skin progression in March 2022, 33 months after commencing treatment with chlormethine hydrochloride gel. (B) Partial response in December 2022, 42 months after commencing treatment with chlormethine hydrochloride gel and bexarotene 225 mg/day.

In March 2022, the patient experienced skin progression in the form of extension of his skin lesions (Figure 1(A)). The patient subsequently discontinued methotrexate and commenced treatment with bexarotene 300 mg/day (half dose) with oral fenofibrate 200 mg/day and L‐thyroxine 50 μg/day. The bexarotene dose was increased to 450 mg/day in June 2022, tapered back to 300 mg/day in July 2022 and then reduced again to 225 mg/day in September 2022 due to the onset of uncontrolled dyslipidaemia.

In December 2022, he achieved a good partial response (PR) to treatment (Figure 1(B)) with the combination of daily chlormethine hydrochloride and bexarotene 225 mg/day, and in November 2023, he underwent surgery for the removal of superficial spreading melanoma (Clark I) localised to the skin of the thorax. The patient remained on the same treatments until March 2024 and has maintained his skin response without any chlormethine hydrochloride gel‐related side effects.

### 2.2. Case 2

**FIGURE 2:**
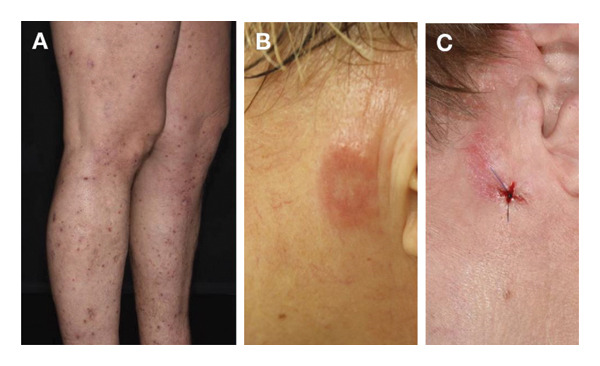
Case 2 clinical images. (A) Folliculotropic mycosis fungoides with keratosis pilaris‐like lesions on the limbs and scratch decorations. (B) Plaque on her left cheek after treatment of a flat tumour nodule with potent topical steroids. Before initiation of a chlormethine hydrochloride and mometasone furoate therapy. (C) Complete response with residual eczema (histologically confirmed) after 10 weeks of treatment with chlormethine hydrochloride gel, mometasone furoate and bexarotene 300 mg/day.

Case 2 reports a 53‐year‐old Caucasian female suffering from folliculotropic MF since 2010 (T2N0M0B0, ISCL/EORTC initial stage IB). She presented with keratosis pilaris–like lesions on her trunk and limbs with severe pruritus (Figure 2(A)). She initially received topical steroids, ultraviolet B‐311 (UVB), psoralen and ultraviolet A (PUVA) (bath) and acitretin 30 mg/day from May 2014 until February 2015 and several antipruritic drugs, such as antihistamines, pregabalin 150 mg/day and mirtazapine 7.5–15 mg/day.

Due to a lack of improvement of the skin lesions, bexarotene (150–300 mg/day) was started in 2017 alongside L‐thyroxine up to 150 μg/day, fenofibrate 200 mg/day and omega‐3 fatty acids. The skin lesions subsequently improved, and the pruritus was reduced.

In October 2021, she developed a flat tumour on her left cheek (T3N0M0B0, ISCL/EORTC stage IIB) which was a large cell transformation and did not respond well to potent topical steroids (Figure 2(B)). In November 2021, based on her doctor’s decision, chlormethine hydrochloride 160 μg/g gel was therefore started every 2 days alternating with mometasone furoate 0.1% w/w cream, which was well tolerated. Systemic treatment with bexarotene was also continued at a dosage of 300 mg/day. After 10 weeks of treatment, the lesion was flattened and reduced in size. In March 2022, chlormethine hydrochloride was stopped, but topical steroids were continued due to residual eczema (Figure 2(C)), which was confirmed by histology.

As blood lipids were significantly elevated, bexarotene was stopped in March 2022 and the patients received 12 cycles of resminostat as part of the Remain study. As findings did not improve, and at the patient’s request, the study treatment was terminated in September 2022 and 5 cycles of extracorporeal photopheresis were administered. In the preauricular region, a flat tumour nodule developed again, which was treated with local radiotherapy (2 × 4 Gy) in January 2023.

Because of further progression and a high rate of CD30‐positive tumour cells in hair follicles, 3 cycles of brentuximab vedotin 1.8 mg/kg body weight were initiated from February to March 2023. As further progression was observed under brentuximab vedotin, treatment was switched to mogamulizumab 1.0 mg/kg body weight from April to May 2023. However, further progression and severe pruritus occurred and bexarotene 150 mg/day was reintroduced in May 2023. A plaque on the forehead was treated from March to August 2023 with chlormethine hydrochloride 160 μg/g gel in the same way as described above and in combination with mogamulizumab 1.0 mg/kg body weight, followed by a combination with bexarotene 150 mg/day. The topical treatment was well tolerated but discontinued by the patient. Local radiotherapy (2 × 4 Gy) was administered in October 2023. Due to stable disease, total skin electron beam therapy was added in August 2024, resulting in partial remission at the time of manuscript preparation.

### 2.3. Case 3

Case 3 describes a 69‐year‐old Caucasian male with Fitzpatrick skin Type II who was diagnosed with MF (T2bN2bM0B0, ISCL/EORTC Stage IIA) in October 2015. The patient first received bath–PUVA and then commenced interferon‐alpha (IFN‐α) 9 × 10^6^ IU three times a week in April 2016. Due to progressive disease, bexarotene 300 mg/day was added to a reduced dose of IFN‐α (3 × 10^6^ IU three times a week) in May 2018. Because of further disease progression, bexarotene was discontinued in April 2019 and local radiation therapy (10 × 3 Gy) was administered to an infiltrated MF plaque left in the inguinal. Following radiation therapy, IFN‐α 3 × 10^6^ IU was once again given as monotherapy three times a week until March 2020, which was then discontinued due to progression to a tumour left inguinal (T3N2bM0B0, ISCL/EORTC Stage IIB).

Starting in April 2020, the patient received 8 doses of brentuximab vedotin every 3 weeks, with the first two doses at 1.8 mg/kg. At the second dose, the patient presented an infusion reaction, resulting in the immediate discontinuation of the infusion. From the third dose onwards, brentuximab vedotin was therefore administered at a dose of 1.2 mg/kg. Due to a lack of efficacy, treatment was terminated in September 2020.

In November 2020, mogamulizumab 1 mg/kg monotherapy once a week was initiated, but stopped temporarily after the 4^th^ dose due to lymphopenia. In May 2022, mogamulizumab was discontinued, when the patient received local radiation therapy (10 × 3 Gy) to his left lower arm. Due to progressive disease, he was switched to subcutaneous methotrexate in July 2022 (starting at a dose of 7.5 mg for 2 months, then 15 mg thereafter) and achieved partial remission. However, a left inguinal skin plaque became erythematous some time after radiation therapy.

**FIGURE 3:**
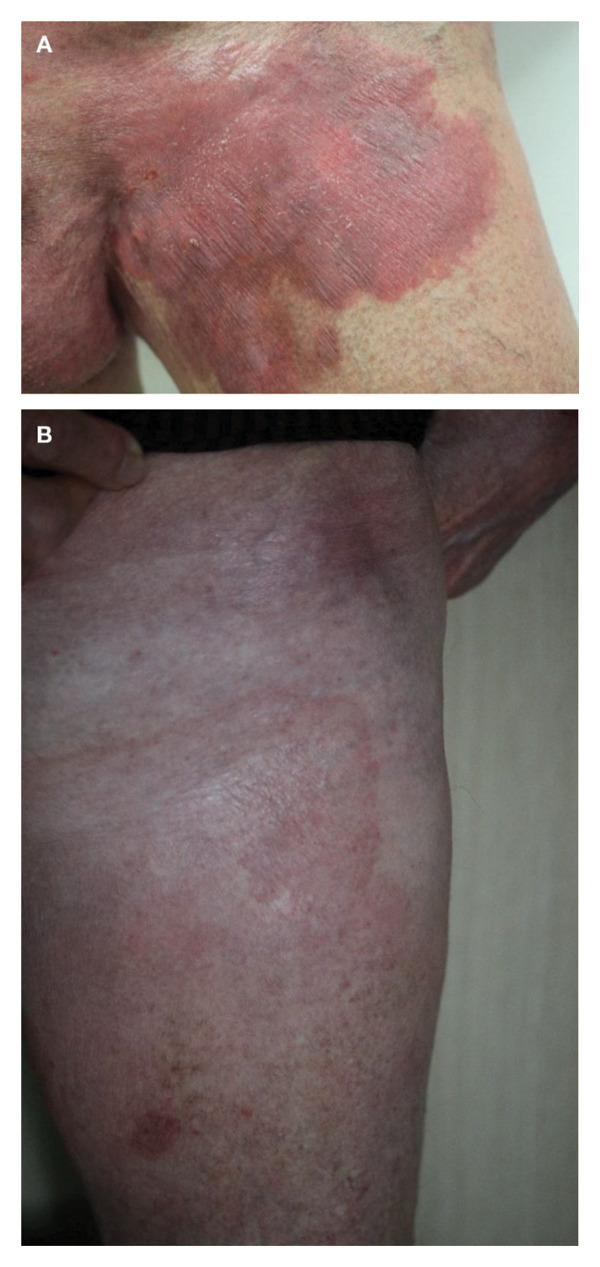
Case 3 clinical images. (A) Erythematous skin plaque located in the left groin region following radiation therapy, shortly before treatment start with chlormethine gel. (B) Improvement with postinflammatory hyperpigmentation following treatment with chlormethine hydrochloride and mometasone furoate, photograph taken 12 months after treatment initiation with chlormethine gel.

Based on the doctor’s decision, chlormethine hydrochloride 160 μg/g gel was commenced in May 2023 (Figure 3(A)), 2 days/week with mometasone furoate 0.1% w/w cream on the following day, and after two weeks of therapy, treatment was intensified to chlormethine hydrochloride 160 μg/g every second day followed by mometasone furoate on the following day. Shortly after dose escalation, the patient experienced erythema and new plaque erosions in treated skin. Chlormethine hydrochloride treatment was therefore interrupted, and the patient continued with mometasone furoate. After skin healing, chlormethine hydrochloride was reinstated at a dosing frequency of 2 days/week with mometasone furoate on the following day. By May 2024, the plaque slowly improved and healed (Figure 3(B)).

At the time of manuscript preparation, the patient was in complete remission and was receiving ongoing treatment with systemic methotrexate 15 mg/week and chlormethine hydrochloride gel 1 day/week followed by mometasone furoate to prevent new infiltration. The treatment has been well tolerated.

### 2.4. Case 4

Case 4 was a 56‐year‐old Caucasian male with a long‐standing history of MF (T3N0M0B0, ISCL/EORTC Stage IIB; primary diagnosis 1995) who had received a range of treatments over a period of 29 years, including skin‐directed therapies (topical steroids, localised radiation) and systemic treatment options (IFN‐α, methotrexate and brentuximab vedotin).

**FIGURE 4:**
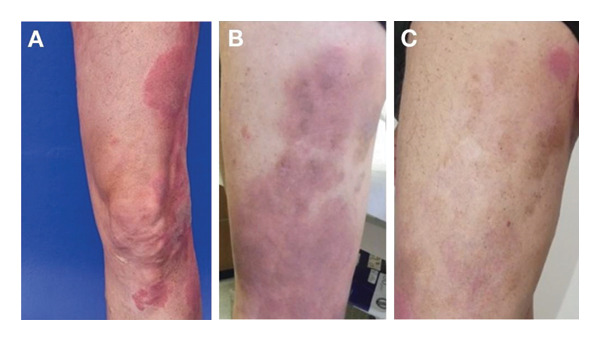
Case 4 clinical images. (A) Infiltrated, treatment‐resistant plaques on the left thigh refractory to bexarotene 100 mg/m^2^/day and topical steroid treatment. (B) Improvement with postinflammatory hyperpigmentation on the left leg following an inflammatory reaction to chlormethine hydrochloride gel. (C) Subsequent stable disease with no new manifestations of lymphoma at 12‐month follow‐up in response to bexarotene.

At presentation in 2019, he was receiving a reduced dose of bexarotene (100 mg/m^2^/day) due to toxicities, alongside topical steroids. He exhibited a mixed response to this treatment regimen after 3 months of treatment, with undulating skin lesions (intermittent amelioration followed by deterioration), including infiltrated plaques on the left thigh that were resistant to treatment (Figure 4(A)). No signs of folliculotropism were present at that site. To address the skin manifestations on the leg, chlormethine hydrochloride 160 μg/g gel was commenced 7 days/week for a period of 6 weeks in addition to bexarotene 100 mg/m^2^/day and topical steroids.

Chlormethine gel was applied to the incremental area until inflammation occurred at the application site; afterwards, the therapy was discontinued due to skin reaction. The inflammatory reaction on the patient’s left leg healed with postinflammatory hyperpigmentation within 4 weeks and partial local remission of prior lymphoma manifestations at the treated site (i.e., the left leg) (Figure 4(B)). The patient achieved stable disease lasting 8 months under continued bexarotene treatment.

At 12‐month follow‐up in October 2020, no new or residual manifestations of lymphoma were present on the patient’s left leg (i.e., the treatment site of chlormethine gel) (Figure 4(C)). However, at the time of manuscript preparation, the patient had undergone progressive disease with the need for subsequent treatment options such as radiation, gemcitabine and mogamulizumab.

### 2.5. Case 5

Case 5 describes a 48‐year‐old Caucasian male who was diagnosed in 2018 with MF (T3N3cM0B0, ISCL/EORTC Stage IVA1) with large cell transformation. The patient initially received various skin‐directed therapies including topical corticosteroids, intralesional triamcinolone and three sessions of localised radiotherapy.

Starting in November 2018, the patient received first‐line therapy with methotrexate 15 mg/week, but due to no response, methotrexate treatment was discontinued after 3 months. The patient subsequently received IFN‐α 3 × 10^6^ IU three times a week in June and July 2019, which resulted in a PR. As IFN‐α was no longer available, the patient was switched to bexarotene (up to 300 mg/m^2^) from July 2019 to March 2020, which initially led to a PR but was followed by the detection of tumour nodules. Therefore, brentuximab vedotin 1.8 mg/kg was administered every 3 weeks from March to July 2020 (6 cycles) and from February 2021 to April 2021 (2 cycles). In May 2021, whole skin electron therapy was performed; however, no response to the treatment was observed.

The patient received pegylated liposomal doxorubicin 20 mg/m^2^ every 2 weeks in July 2021 (2 cycles) but subsequently experienced progressive disease with lymph node involvement, resulting in the discontinuation of doxorubicin. He then received gemcitabine 1000 mg/m^2^ every 2 weeks in August 2021. Following the development of progressive lymphoedema of the legs with lymph node involvement, radiation of the inguinal and axillary lymph nodes was performed. Subsequent treatment with etoposide 100 mg/m^2^ and ifosfamide 1500 mg/m^2^ (1 cycle) for 3 consecutive days every 3 weeks in October 2021 resulted in a complete response. He underwent allogeneic stem cell transplantation in November 2021, demonstrating a sustained complete response.

**FIGURE 5:**
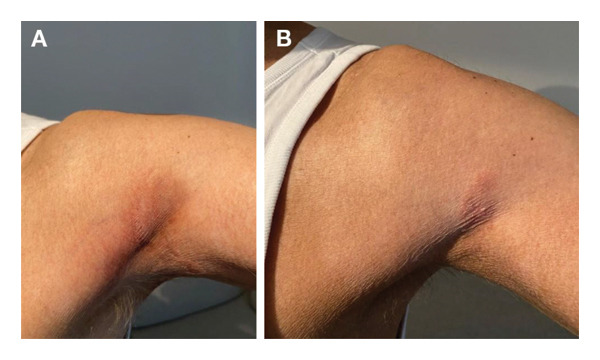
Case 5 clinical images. (A) Recurrence in the left axilla after first allogeneic stem cell transplantation. (B) Good tumour control following donor lymphocyte infusion and chlormethine hydrochloride gel.

In February 2022, the patient experienced disease recurrence (Figure 5(A)) and was subsequently treated with a donor lymphocyte infusion and chlormethine hydrochloride 160 μg/g gel once a day, which achieved good tumour control in June 2022 (Figure 5(B)). However, the patient died at the beginning of 2023 due to lymph nodal progression.

### 2.6. Case 6

Case 6 was a 60‐year‐old Caucasian male with a long history of MF (T4N0M0B0, ISCL/EORTC Stage IIIA) in 2019. He had been diagnosed in 1978 and after receiving PUVA in 1979 achieved complete remission.

**FIGURE 6:**
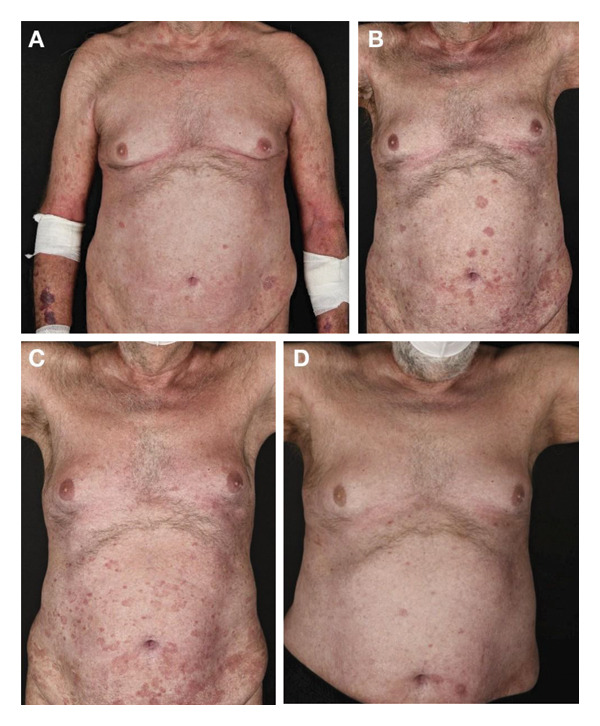
Case 6 clinical images. (A) Recurrence on the torso while receiving bexarotene and extracorporeal photopheresis in March 2021. (B) Increase in recurrence in April 2021. (C) Unmasking of the lesions in September 2021 following the addition of chlormethine hydrochloride gel to the treatment regimen. (D) Response in January 2022.

The patient experienced recurrence in 1999, which was categorised as advanced disease. He was treated with decreasing doses of systemic cyclosporine treatment, which led to improvement, but was discontinued in 2001 due to renal insufficiency. Another recurrence was detected through thorough examination in 2012. The patient therefore received PUVA in April 2013 and bexarotene monotherapy 300 mg/m^2^ from 2013 to 2019, after which he initiated bexarotene 300 mg/m^2^ combined with extracorporeal photopheresis. However, due to disease progression from March 2021 (Figure 6(A)) to April 2021 (Figure 6(B)), treatment was escalated with the addition of once‐daily chlormethine hydrochloride 160 μg/g gel to bexarotene and extracorporeal photopheresis. By September 2021, this treatment regimen had led to an unmasking of the lesions (Figure 6(C)) followed by a response in January 2022 (Figure 6(D)). At the last visit in August 2024, the patient was in near complete remission.

### 2.7. Case 7

Case 7 describes a 54‐year‐old Caucasian female diagnosed with MF (T2bN0M0B0b, ISCL/EORTC Stage IB; gamma/delta phenotype) in 2019, with skin patches and plaques recurring since 2004. The patient received narrowband UVB phototherapy and acitretin 10 mg/day starting in 2019, but the treatment was discontinued in 2022 due to treatment‐emergent effluvium.

**FIGURE 7:**
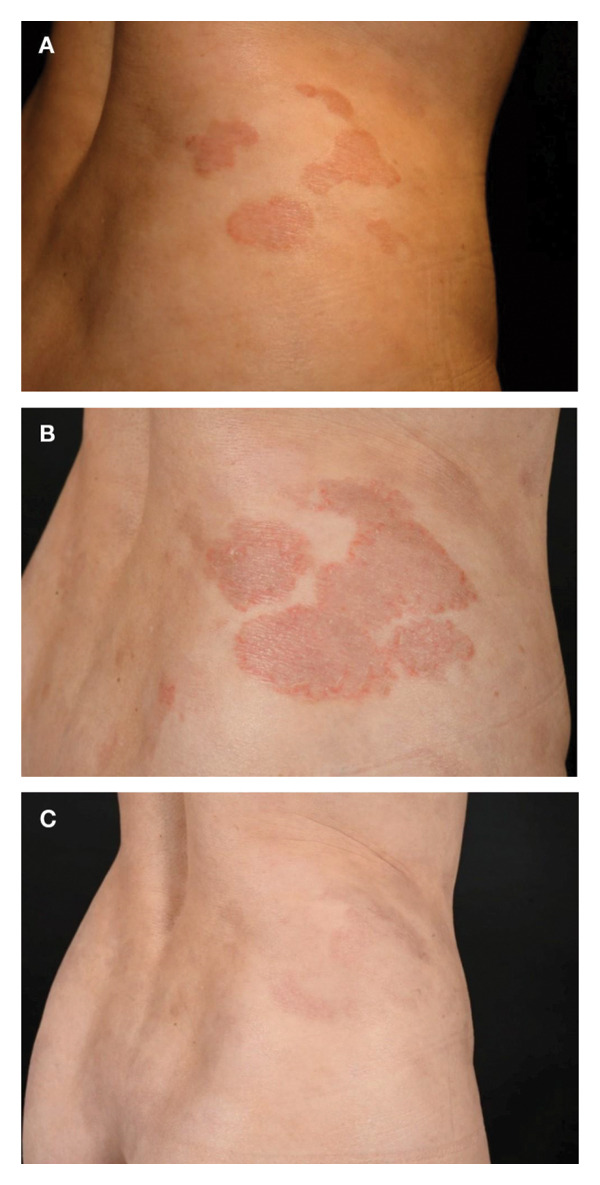
Case 7 clinical images. (A) Skin patches and plaques at presentation in October 2022. (B) Progression of skin patches and plaques after initiation of chlormethine hydrochloride gel monotherapy. (C) Partial response following combination therapy of chlormethine hydrochloride gel and IFN‐α 2a. IFN‐α, interferon‐alpha.

In October 2022 (Figure 7(A)), monotherapy with chlormethine hydrochloride 160 μg/g gel once daily was initiated but resulted in an insufficient response by May 2023 (Figure 7(B)); the treatment was well tolerated. IFN‐α 2a 135 μg/week was subsequently prescribed as an add‐on to chlormethine hydrochloride gel therapy. After three months, a good treatment response was achieved (Figure 7(C)), but due to fatigue and flu‐like symptoms, the dose of IFN‐α 2a was reduced to 90 μg/week. By September 2023, further clearance of skin plaques was noted, with only minimal residual disease left. However, while fatigue and flu‐like symptoms improved, effluvium emerged as a new side effect. The patient therefore decided to discontinue chlormethine therapy, which resulted in worsening of skin plaques between September and November 2023. In December 2023, the skin status was unchanged, but the patient discontinued therapy with IFN‐α 2a also due to ongoing effluvium.

Due to continuous disease progression with newly evolving patches and plaques, combination therapy of chlormethine hydrochloride gel once daily and IFN‐α 2a 135 μg/week was restarted in February 2024. Within a month of resuming the combination treatment, the patient experienced partial remission with clearance of all plaques and improvement in patches, but she paused the application of chlormethine hydrochloride gel again as she was unable to store the gel properly.

From March to May 2024, her condition was stable, but, by July 2024, the skin patches had worsened and new plaques evolved, indicating that IFN‐α 2a monotherapy was insufficient. The patient was therefore prescribed chlormethine hydrochloride once again in combination with IFN‐α 2a 90 μg/week and presented with minimal residual disease and no side effects in September 2024. Due to the permanent unavailability of IFN‐α 2a in Austria starting in October 2024, the patient was switched to ropeginterferon (IFN‐α 2b) 100 μg every two weeks while left on chlormethine hydrochloride once daily. Eight weeks after starting this combination regimen, the patient reported good tolerability and ongoing symptom relief with only a few patches remaining (T1aN0M0B0).

The treatment regimens, best response and skin‐related AEs of all cases are summarised in Table 1.

**TABLE 1:** Summary table of treatment regimens, responses and AEs of all cases.

	Treatment prior to CL combination therapy	CL combination therapy	Best response	Skin‐related AEs
Case 1	• 30 sessions of phototherapy	• SC MTX 15 mg/week and CL 160 μg/g gel 5 days/week • Daily CL and BEX 225 mg/day	• PR	• No AEs reported

Case 2	• Topical steroids, UVB, PUVA (bath), acitretin 30 mg/day and several antipruritic drugs, such as antihistamines, PGB 150 mg/day and MIRT 7.5–15 mg/day • BEX (150–300 mg/day) with LT4 up to 150 μg/day, fenofibrate 200 mg/day and omega‐3 fatty acids	• First round: CL 160 μg/g gel every 2 days alternating with MF 0.1% w/w cream and BEX at 300 mg/day • Second round: CL 160 μg/g gel with MOGA 1.0 mg/kg body weight followed by a combination with BEX 150 mg/day	• CR after first round	• Residual eczema

Case 3	• PUVA (bath) and IFN‐α 9 × 10^6^ IU • BEX 300 mg/day was added to a reduced dose of IFN‐α (3 × 10^6^ IU 3x a week) • Local radiation therapy (10 × 3 Gy) • IFN‐α 3 × 10^6^ IU given as monotherapy 3x a week • BV every 3 weeks at 1.8 mg/kg • MOGA 1 mg/kg monotherapy • Local radiation therapy (10 × 3 Gy) • SC MTX	• CL 160 μg/g gel, 2 days/week with MF 0.1% w/w cream on the following day and after 2 weeks of therapy, CL 160 μg/g gel every second day followed by MF on the following day • At remission, systemic MTX 15 mg/week and CL 1 day/week followed by MF	• CR	• Erythema and new plaque erosions in treated skin

Case 4	• Skin‐directed therapies (topical steroids, localised radiation) and systemic treatment options (IFN‐α, MTX and BV) • BEX (100 mg/m^2^/day), alongside topical steroids	• CL 160 μg/g gel 7 days/week for 6 weeks in addition to BEX 100 mg/m^2^/day and topical steroids	• PR	• Inflammation, resulting in discontinuation

Case 5	• Various skin‐directed therapies including topical corticosteroids, intralesional triamcinolone and 3 sessions of localised radiotherapy • First‐line therapy with MTX 15 mg/week • IFN‐α 3 × 10^6^ IU 3x a week • BEX (up to 300 mg/m^2^) and then BV 1.8 mg/kg every 3 weeks • Whole skin electron therapy • PLD 20 mg/m^2^ every 2 weeks and GEM 1000 mg/m^2^ every 2 weeks • Radiation of the inguinal and axillary lymph nodes • VP‐16 100 mg/m^2^ and IFO 1500 mg/m^2^ (1 cycle) for 3 consecutive days every 3 weeks • Allogeneic stem cell transplantation	• Donor lymphocyte infusion and CL 160 μg/g gel once a day	• Good tumour control	• Not reported

Case 6	• PUVA • Systemic cyclosporine treatment • PUVA and BEX monotherapy 300 mg/m^2^ • BEX 300 mg/m^2^ combined with ECP	• Once‐daily CL 160 μg/g gel with BEX 300 mg/m^2^ and ECP	• PR (near CR)	• Not reported

Case 7	• Narrowband UVB phototherapy and ACI 10 mg/day	• First two rounds: CL 160 μg/g gel once daily and IFN‐α 2a 135 μg/week • Third round: CL combined with IFN‐α 2a 90 μg/week and then switched to IFN‐α 2b 100 μg every 2 weeks while left on CL once‐daily	• PR	• Fatigue, flu‐like symptoms and effluvium after the first round

Abbreviations: ACI, acitretin; AE, adverse event; BEX, bexarotene; BV, brentuximab vedotin; CL, chlormethine hydrochloride; CR, complete response; ECP, extracorporeal photopheresis; GEM, gemcitabine; IFN‐α 2b, ropeginterferon; IFN‐α, interferon‐alpha; IFO, ifosfamide; LT4, L‐thyroxine; MF, mometasone furoate; MIRT, mirtazapine; MOGA, mogamulizumab; MTX, methotrexate; PGB, pregabalin; PLD, pegylated liposomal doxorubicin; PR, partial response; PUVA, psoralen and ultraviolet A; SC, subcutaneous; SD, stable disease; UVB, ultraviolet B‐311; VP‐16, etoposide.

## 3. Discussion

Our real‐world patient case series demonstrated that chlormethine hydrochloride gel as a partner in combination therapy regimens is effective for disease control evidenced by clinical clearance and/or reduction of skin lesions in patients with Stage I–IV MF. These combination therapies included chlormethine hydrochloride gel with either skin‐directed and/or systemic therapies, predominantly bexarotene. In all but one of the patients described here, chlormethine hydrochloride combination treatment was associated with plaque reduction, stable disease or a partial/complete response. One patient who received chlormethine hydrochloride gel in addition to bexarotene and topical steroids (Case 4) experienced inflammation at the application site, resulting in the permanent discontinuation of the gel.

Data from the prospective, real‐world PROVe (PROspective, observational study assessing outcomes, AEs, treatment patterns and QoL in patients diagnosed with MF and treated with chlormethine hydrochloride gel and other therapies) study demonstrated treatment responses, as measured by percentage body surface area, 12 months after treatment initiation in 44%–45% of US patients with MF (*N* = 298) receiving chlormethine gel in combination with other therapies such as topical steroids, phototherapy and oral bexarotene [16]. In addition, a single‐centre, real‐world study in 66 patients with early‐stage MF receiving chlormethine gel documented an objective response rate (ORR) in 50% of patients. Some of these patients received chlormethine gel in combination with topical corticosteroids. The study estimated that median time to achieve a ≥ 50% improvement from baseline was 38.1 weeks (95% confidence interval 24.5–51.7) [17]. The PROVe study also reported a delay in peak response, potentially due to gradual dose increases, highlighting the importance of continued chlormethine treatment [16]. Similarly, a large real‐world study in > 1100 patients with MF reported responses to chlormethine as late as 6 months after treatment initiation, with a complete response taking up to 24 months to achieve in some patients [18]. Indeed, a 2022 expert consensus recommends that chlormethine therapy should not be discontinued too early due to potential delayed responses referring to the late responses reported in some patients [5].

Patients in our case series with the shortest time of chlormethine gel treatment also experienced the least benefit from the topical treatment (Case 4), suggesting the potential benefit of restarting the treatment after the resolution of AEs. Furthermore, a patient who discontinued chlormethine gel therapy on her own (Case 7) experienced worsening of skin patches and plaques while on remaining treatment with IFN‐α 2a and subsequent improvement of the skin lesions after re‐challenge with chlormethine gel therapy in combination with IFN‐α 2a and IFN‐α 2b.

Approximately 10% of the patients enrolled in the PROVe study had Stage IIA–IV MF [16]. These patients were likely using chlormethine gel for local disease control of patches and plaques, which is supported by our own patients, some of whom had received numerous lines of prior therapy and were prescribed chlormethine gel to manage specific/residual skin manifestations. In our series, chlormethine hydrochloride was most commonly coadministered with topical steroids in addition to bexarotene or methotrexate; however, extracorporeal photopheresis, IFN‐α 2a and brentuximab vedotin were also used as combination partners of chlormethine gel. In PROVe, 78% of patients used chlormethine gel in combination with other skin‐directed therapies, and 30% in combination with systemic therapies. The most common of these therapies was topical corticosteroids (60%) and oral bexarotene (16%) [16].

Data from this and other studies indicate that combining chlormethine with other therapies in MF does not cause additional side effects and may improve response rates [16, 18]. Indeed, due to the local mode of action and lack of systemic absorption of chlormethine, it may be particularly suitable for coadministration with systemic therapies. However, the 2022 expert consensus gives no guidance on the use of concomitant therapies other than topical corticosteroids, antiseptics and anti‐infectives due to too little scientific evidence [5].

The different chlormethine combination regimens were generally well tolerated in the cases presented here. The AEs associated with chlormethine gel therapy were inflammation at the application site, pruritus and erythema. Dyslipidaemia was only seen in a patient receiving bexarotene as a treatment combination partner. The reasons for chlormethine discontinuation were documented as follows: plaque erosions and erythema, skin reaction and effluvium. The latter side effect was most likely caused by IFN‐α 2a as it was ongoing after discontinuation of chlormethine gel. Overall, the use of chlormethine gel in combination therapy was not associated with worse AEs than chlormethine gel monotherapy. In the PROVe study, the most common skin‐related AEs were dermatitis, pruritus, skin irritation and erythema, with no serious treatment‐related AEs, supporting our own findings [16]. Similarly, in the randomized Study 201 mentioned earlier, contact dermatitis was the most common skin‐related AE and could be managed with dose adjustments and the use of emollients and antihistamines [14], while the large, multicentre real‐world study (*N* > 1100) reported contact dermatitis in 20%–30% of patients [18]. Notably, a single‐centre, real‐world study in 66 patients with early‐stage MF receiving chlormethine reported no difference in the proportion of patients achieving an ORR between those with versus without dermatitis (37.5% vs. 57.1%; *p* = 0.2) [17]. Data suggest that patients who experience mild‐to‐moderate hypersensitivity reactions after initiation of chlormethine therapy can continue treatment after intervention, and even patients with severe skin reactions may be able to restart treatment at lower dosing frequencies [7]. Indeed, the 2022 expert consensus recommends that chlormethine therapy should be resumed once severe dermatological AEs have subsided [5]. Coadministration of chlormethine with topical steroids can help manage AEs, such that chlormethine therapy may be continued for a longer treatment duration [10]. It was previously reported that coadministration of triamcinolone 0.1% ointment with chlormethine gel does not adversely affect the gel’s efficacy, but, in fact, improves its tolerability by reducing the severity of dermatitis [19], an AE commonly observed in several of our cases. This may be significant as discontinuations due to AEs seem to occur soon after commencing treatment [20]. To support the optimal use of chlormethine gel, patients should be educated on the appropriate application process, the appropriate use of topical steroids and the management of adverse skin reactions [10].

In conclusion, our real‐world case series demonstrates the efficacy and tolerability of chlormethine hydrochloride gel, in combination with other topical and systemic therapies, to reduce the severity of skin lesions in patients with Stage I–IV MF in clinical practice, and illustrates how responses may be achieved in patients who have previously received multiple lines of therapy. AEs associated with chlormethine gel were generally mild‐to‐moderate in intensity and could generally be managed with temporary dose interruption or appropriate topical interventions.

## Author Contributions

All authors were involved in the clinical care of their respective patients and contributed to the writing and editing of the manuscript.

## Funding

Funding for editorial assistance was provided by Recordati Rare Diseases. Open Access funding enabled and organized by Projekt DEAL.

## Disclosure

All authors read and approved the final manuscript.

## Ethics Statement

Written informed consents were obtained from the patient or next of kin for publication of each individual case and any accompanying images. Copies of the written consents are available for review by the associate editor of this journal. This retrospective case series was conducted in compliance with Good Clinical Practice guidelines and in accordance with the Declaration of Helsinki (1964).

## Conflicts of Interest

Gabor Dobos received honoraria and participated in Advisory Boards for Recordati Rare Diseases, Helsinn, Kyowa Kirin, Mallinckrodt Therakos and Takeda.

Christina Mitteldorf received consultant and/or paid speaker fees and/or received travel reimbursement from Takeda, Recordati Rare Diseases, Kyowa Kirin and Stemline Therapeutics.

Johanna Hoffmann declares no conflicts of interest.

Kai‐Christian Klespe participated in Advisory Boards and received honoraria and travel grants from Bristol‐Myers Squibb, Kyowa Kirin, Novartis, Pierre Fabre, Recordati Rare Diseases, Sun Pharma and Vetter Pharma.

Marion Wobser received funding for congress participation and consulting fees by Kyowa Kirin, Takeda, Recordati Rare Diseases and Stemline Therapeutics.

Adèle de Masson participated in Advisory Boards and received honoraria from Recordati Rare Diseases, Helsinn, Takeda and Kyowa Kirin.

Constanze Jonak received speaker and/or consultancy fees, and/or travel grants from Recordati Rare Diseases, Takeda, Kyowa Kirin and Stemline Therapeutics and received investigator grants from Innate Pharma and 4SC paid to her institution.

## Data Availability

Data are available upon request from the authors.
